# Event and Comment

**Published:** 1908-02-15

**Authors:** 


THE
### DENTAL REGISTER.

Vol. LXII. February 15, 1908. No. 2.

### EVENT AND COMMENT.

### Misdirected Energy.

How much of our precious time we waste in doing needless things, or in finding out how to do things properly, we shall never know. It is only the man who does routine work of a mechanical character that really seems to use up all his time in accomplishing results. In the practice of dentistry we meet at almost every step some detail of treatment that is new, and there are few callings that demand so versatile minds and hands as ours. It takes a lifetime of practice to enable one to have even tolerably good judgment. In these latter days, when so many new technical and theropeutic procedures are springing up every day, it is really discouraging for the average man to attempt to keep up with every new thing. Our journals and society meetings are doing a noble work in their efforts to cultivate the profession, and the colleges are making strenuous efforts to keep their teaching up to date; and some are even doing what they can to keep the practitioners up by giving short courses on up-to-date methods of practice and scientific advancement. This is is good, and ought to mak us proud of our profession and the men who are making it great.

We recently encountered a situation in one of our large cities that is so perfectly absurd, if not illogical and absolutely wicked, that we cannot refrain from making a protest against it. It seems that several years ago in this city a great dental convention was to be held, and the local dentist of course were expected to make the local arrangements; and in their zeal to do the best thing possible a division of opinion arose, and the profession lined up on two sides in battle array. The controversy grew and became so fierce that it has apparently split the profession of that city into two parties almost equally divided, and with little prospect short of Divine intervention, that there ever will be an amicable adjustment and the establishment of harmony and a proper *esprit de corps.*

Now, what good can ever possibly come of such a condition of affairs? The cause of the difference is one of personal opinion and is purely selfish, as there is absolutely no principle worth naming at stake. Some of the most active participants will not recognize each other on the street. A continuous propaganda of abuse and bad actions keeps the nettles stinging, and if anything, time does not seem to be healing the wounds at all If one-hundredth part of the time, thought and energy that is being used to keep this feud alive was properly directed, under the guidance of a forgiving spirit to the adjustment of their differences, how much more happy and prosperous would be all who are now strenuously wasting time, temper, health, and it may be the hope of or desire for a happy hereafter.

It is a shame that intelligent men of good intentions will allow mere personal pique to stand between them and their opportunity to fulfill the object for which they were born, if it is true that ours is a calling—and only those who were born for the work are engaged in it. There is still a great work to be done by our profession, though it does seem that the vast majority see no occasion to exercise themselves to help solve the great problem of preventing decay of the teeth. We may not use every dentist in accomplishing this vast undertaking, but we do need unmistakably a united profession that will support a high ideal and proper sentiment, which shall call out our best minds and hearts to strive and sacrifice for the accomplishment of the object before us. We can't afford to have dissatisfaction, because it kills enthusiasm and who knows but it may be the means of turning aside the very man who should have led us far toward the solution of our problems. This case we have just cited is not the only one of record, or in fact at the present time; but we do hope the wisdom of intelligent men will override selfishness and absurdity, and that such absurdity shall soon pass away.

